# Anti-Inflammatory Mechanisms of Apolipoprotein A-I Mimetic Peptide in Acute Respiratory Distress Syndrome Secondary to Sepsis

**DOI:** 10.1371/journal.pone.0064486

**Published:** 2013-05-14

**Authors:** Oleg F. Sharifov, Xin Xu, Amit Gaggar, William E. Grizzle, Vinod K. Mishra, Jaideep Honavar, Silvio H. Litovsky, Mayakonda N. Palgunachari, C. Roger White, G. M. Anantharamaiah, Himanshu Gupta

**Affiliations:** 1 Department of Medicine, University of Alabama at Birmingham, Birmingham, Alabama, United States of America; 2 Medicine Service, United States Department of Veterans Affairs Medical Center, Birmingham, Alabama, United States of America; 3 Department of Pathology, University of Alabama at Birmingham, Birmingham, Alabama, United States of America; 4 Department of Medicine, Department of Biochemistry and Molecular Genetics, University of Alabama at Birmingham, Birmingham, Alabama, United States of America; D'or Institute of Research and Education, Brazil

## Abstract

Acute respiratory distress syndrome (ARDS) due to sepsis has a high mortality rate with limited treatment options. High density lipoprotein (HDL) exerts innate protective effects in systemic inflammation. However, its role in ARDS has not been well studied. Peptides such as L-4F mimic the secondary structural features and functions of apolipoprotein (apo)A-I, the major protein component of HDL. We set out to measure changes in HDL in sepsis-mediated ARDS patients, and to study the potential of L-4F to prevent sepsis-mediated ARDS in a rodent model of lipopolysaccharide (LPS)-mediated acute lung injury, and a combination of primary human leukocytes and human ARDS serum. We also analyzed serum from non-lung disease intubated patients (controls) and sepsis-mediated ARDS patients. Compared to controls, ARDS demonstrates increased serum endotoxin and IL-6 levels, and decreased HDL, apoA-I and activity of anti-oxidant HDL-associated paraoxanase-1. L-4F inhibits the activation of isolated human leukocytes and neutrophils by ARDS serum and LPS *in vitro*. Further, L-4F decreased endotoxin activity and preserved anti-oxidant properties of HDL both *in vitro* and *in vivo*. In a rat model of severe endotoxemia, L-4F significantly decreased mortality and reduces lung and liver injury, even when administered 1 hour post LPS. Our study suggests the protective role of the apoA-I mimetic peptide L-4F in ARDS and gram-negative endotoxemia and warrant further clinical evaluation. The main protective mechanisms of L-4F are due to direct inhibition of endotoxin activity and preservation of HDL anti-oxidant activity.

## Introduction

Sepsis is frequently associated with acute respiratory distress syndrome (ARDS), which is a leading cause of morbidity and mortality in critically ill patients [1]. Lipopolysaccharide (LPS), an endotoxic component of the outer membrane of gram-negative bacteria, mediates many of the toxic effects associated with sepsis, including inflammation and oxidative stress [2], [3]. LPS activates Toll-like receptors present on leukocytes and endothelium leading to stimulation of NF-κB and mitogen-activated protein kinase pathways that cause synthesis and liberation of various pro-inflammatory mediators [4]–[6]. TNF-α and IL-6 are important mediators of the LPS-activated inflammatory cascade leading to liver injury and acute lung injury (ALI) [7]–[9]. A high plasma IL-6 level serves as a poor prognostic indicator for sepsis-related ARDS/ALI [10]–[13].

Plasma lipoproteins play an important role in LPS neutralization by binding to circulating LPS and transporting it to the liver where it gets metabolized and excreted in the bile [14]–[16]. High density lipoprotein (HDL) possesses the highest binding capacity for LPS compared to other lipoproteins [15], [17]. Septic patients have decreased plasma HDL levels [18]. In sepsis, HDL undergoes remodeling and is converted to an acute-phase lipoprotein with pro-inflammatory and pro-oxidant properties [18]–[20]. Plasma levels of anti-inflammatory apolipoprotein A-I (apoA-I), the major protein constituent of HDL, and anti-oxidant enzyme paraoxonase 1 (PON1), are also reduced by sepsis [18], [20]–[22]. Decreased activity of PON1 in septic patients is associated with oxidative stress and inflammation progress [20]–[22]. Raising plasma HDL is associated with reduction in LPS-induced inflammation in humans [23] and improved survival in experimental animal models [24]. Reconstituted HDL attenuates ALI in endotoxemic rats [25]. Administration of human apoA-I also inhibits ALI and improves survival in endotoxemic mice [26]. Although raising plasma HDL represents an important goal for sepsis treatment [20], obtaining therapeutic quantities of HDL/apoA-I is impractical. Moreover, the mechanisms for the protective effects of apoA-I in ARDS are not well understood.

To develop an alternative approach for HDL therapy, we have studied apoA-I mimetic peptides [27], [28]. The 4F peptide, composed of 18 amino acids, mimics the helical repeating domains of apoA-I and has strong anti-atherogenic and anti-inflammatory properties [27]–[30]. We previously reported that 4F reduces cardiovascular dysfunction and improves survival in rodent models of sepsis [31], [32]. It is hypothesized that 4F may exert similar protective effects against tissue injury induced by gram-negative sepsis in humans, including sepsis-mediated complication such as ARDS/ALI. To support this hypothesis and study mechanisms, we utilized a combination of primary human leukocytes, a rodent model of LPS-mediated ALI and human ARDS serum. Some of the results of these studies have been previously reported in the form of an abstract [33].

## Materials and Methods

### Study approval

All human studies were approved by the University of Alabama at Birmingham (UAB) IRB. All subjects or their representatives (when applicable) provided written informed consent. All procedures involving animals were conducted in accordance with the guidelines of the Care and Use of Laboratory Animals and the National Institutes of Health and approved by the IACUC of the UAB.

### Peptides and LPS

L-4F, composed of the L-amino acid sequence Ac-DWFKAFYDKVAEKFKEAF-NH_2_ was synthesized by the solid phase peptide synthesis method [34]. The scrambled peptide (Sc-4F, Ac-DWFAKDYFKKAFVEEFAK-NH_2_) was synthesized by rearranging the amino acid sequence of L-4F. Sc-4F is unable to form an amphipathic helix and was used as a control for L-4F. Peptide purity was ascertained by mass spectral analysis and analytical high-performance liquid chromatography. LPS (Escherichia coli 026:B6, Sigma-Aldrich) and Bodipy-LPS (Escherichia coli 055:B5, Molecular Probes) was obtained commercially.

### In vitro experiments

#### Patient Populations and Blood Collection

ARDS patients secondary to sepsis (n = 6) and non-lung disease patients (n = 6), who were also intubated and mechanically ventilated, were recruited from the UAB hospital. Patient's age varied from 33 to 69 years old (52.8±3.2 y.o., Table S1). They were either African-American (50%) or Caucasian (50%) and included both male (58%) and female (42%). All subjects carried the diagnosis of ARDS based on accepted diagnostic criteria [35]. Blood samples were collected and centrifuged at 2500 rpm for 15 min, and sera were stored at −80°C for later use. Blood samples from healthy volunteers were collected by venipuncture into heparinized VacutainerTM tubes (Becton Dickinson) or VACUETTE® serum tubes (Greiner Bio-One).

#### *In vitro* experiments with isolated neutrophils

Neutrophils were isolated from peripheral blood of healthy volunteers as previously described [36] by separation on a Ficoll-Histopaque gradient (Sigma-Aldrich, St. Louis, MO). The final suspension of neutrophils contained more than 96% of viable cells as evaluated by trypan blue exclusion. The pre-warmed neutrophils suspensions (37°C, 3.5×10^6^ cells/ml) were incubated in serum from either healthy donors or ARDS patients with or without L-4F (40 μg/ml) or Sc-4F for 1 h at 37°C in 5% CO_2_. Following incubation, neutrophils were isolated by gentle centrifugation and stained for measuring CD11b expression, and cell-free supernatant was analyzed for levels of MPO and endotoxin and activity of HDL-associated PON1.

#### *In vitro* measurement of oxidative stress using lucigenin-amplified chemiluminescence

Fresh human leukocytes were collected from the blood of healthy volunteers by aspiration of a buffy coat. Any remaining red blood cells in buffy coat were lysed; leukocytes were then centrifuged and twice washed in PBS. Leukocytes (1–2×10^6^ cell/ml) were incubated in serum from either healthy donor or ARDS patients in the absence and presence of 40 µg/ml L-4F. Lucigenin (10 μM) was added 2 min before adding L-4F and onset of measurements. Lucigenin-amplified chemiluminescence was measured for 3 h as a marker of superoxide formation [37]. Area under curves (AUC) of relative photon emission (RPE) over time were calculated and compared.

#### *In vitro* experiments with Bodipy-LPS

To investigate interaction of LPS with blood cells in the presence and absence of L-4F, fluorescent Bodipy-LPS (E. Coli 055:B5, Molecular Probes) with or without L-4F was incubated in the whole blood for 30 min. White blood cells were isolated from the blood by aspiration of a buffy coat as described above and then analyzed on Becton Dickinson FACSCalibur flow cytometer for Bodipy signal (Ex488/Em530). White blood cells were gated according to physical characteristics (FSC/SSC) with assistance of an experienced scientist from flow-cytometry core facility. We measured Bodipy mean fluorescent intensity (MFI) in each major leukocyte subtypes (granulocytes, monocytes and lymphocytes). Changes in Bodipy-LPS and L-4F-induced MFI, relative to background MFI in control cells (no Bodipy-LPS) were analyzed. Biological activity of Bodipy-LPS was determined by incubating in the whole human blood and following measurements of cytokine production.

#### *In vitro* experiments with human blood

To 1 ml of blood from a healthy volunteer either: 1) saline; 2) L-4F 40 µg/ml; 3) Sc-4F 40 µg/ml; 4) LPS 1 µg/ml (E. coli 026:B6, Sigma-Aldrich); 5) LPS and L-4F; or 6) LPS and Sc-4F was added and incubated for 12 h at 37°C in 5%CO_2_. The blood was then centrifuged at 4°C for 20 minutes at 300 g. Collected plasma aliquots were frozen (for cytokine and endotoxin analysis) or kept at 4°C.

#### Measurement of molecular interaction of L-4F with LPS using Circular Dichroism spectroscopy

Circular dichroism (CD) spectroscopy of L-4F 100 μg/ml with and without LPS 1000 μg/ml was performed in PBS. The CD spectra were recorded using JASCO J-815 CD spectrometer (JASCO model PTC-423S/15). The CD spectra were measured from 250 to 195 nm every 1 nm with 4 s averaging per point and a 2 nm bandwidth. A 0.1 cm path length cell was used for obtaining the spectra. The CD spectra were signal averaged by adding four scans, baseline corrected, and smoothed. All the CD spectra were recorded at 37°C. The mean residue ellipticity, [Θ]MRE (deg cm^2^ dmol^−1^), was calculated using the following equation: [Θ]MRE  =  (MRW × Θ)/(10cl), where, MRW is the mean residue weight (molecular weight of the peptide divided by the number of amino acids in the peptide), Θ is the observed ellipticity in degrees, c is the concentration of the peptide in grams per milliliter, and l is the path length of the cell in centimeters. MRE was measured at 222 nm as previously published [34], [38].

#### Flow cytometry for cd11b detection

Following incubation with patient serum (see above), neutrophils were washed with PBS and incubated with BSA-based FACS buffer for 15 min at room temperature. Neutrophils were again washed with PBS and were incubated in BSA-based FACS buffer with mouse anti-human cd11b (activation epitope) allophycocyanin (APC) (eBioscience) for 30 min at 4°C. After washing with PBS, the cells were fixed in 2% paraformaldehyde. Samples were controlled for by corresponding negative controls (including unstained samples and mouse IgG1 K Isotype Control APC, eBioscience). CD11b expression (based on mean fluorescent intensity) was measured by flow cytometry using FACS Calibur (Becton Dickinson). Results were normalized to control as a percent change.

### In vivo rat experiments

#### Experimental design

Adult male Sprague-Dawley rats, weighing 322±8 g (Charles Rivers, Wilmington, MA), were used throughout the study. Prior to experiments, the rats were acclimatized for one week in a 12 h light/dark cycle with free access to food and water. Nonfasted rats underwent either injection of LPS (Escherichia coli 026:B6, Sigma-Aldrich, 30 mg/kg, i.p.) or Saline (1 ml). One hour after LPS injection, rats were randomized to receive either L-4F (10 mg/kg, i.v.) or saline (1 ml). After 6 h, rats were euthanized and either lungs or bronchoalveolar lavage fluid (BALF) and liver were collected. In a separate experiment, L-4F was injected either concurrently or one hour after LPS administration. Cumulative survival was evaluated at 24 h.

#### Tissue collection protocol

After ketamine/xylazine anesthesia, abdominal cavity and chest were opened and heparinized blood sample was collected from the right ventricle, followed by exsanguination via the abdominal aorta. The right liver lobe was cut and frozen in liquid nitrogen, and then lungs or broncholaviolar lavage fluid (BALF) was collected. In the lung group, the cannula was inserted into the lung artery via the right ventricle, and the lung vascular system was slowly perfused with 10 ml of cold PBS, followed by perfusion with 10 ml of cold 4% buffered formalin. The lungs were dissected and placed in 4% buffered formalin for 18 h. In the BALF group, the cannula was fixed in the trachea, and the lungs were lavaged gently, via this cannula, to avoid tissue rupture. Lavage fluid (5 mL) was slowly injected into the lung using a syringe and then sucked out again. The operation was repeated 3 times with fresh cold PBS. Total BALF of 15 ml was collected and centrifuged at 300× g for 10 min at 4°C. The supernatants were kept frozen at −80°C until assayed, and the cell pellet was resuspended in sterile PBS for cell counting.

#### Histological analysis

Paraffin-embedded lung and frozen liver tissues were sectioned at 5 µm and stained with hematoxylin and eosin for morphological analysis. Sections of lungs (5–8 rats/group) and liver (4 rats/group) were analyzed using standard histological techniques and the level of tissue inflammation/damage was subjectively assessed by blinded experienced pathologists. Lung microscopy was performed using Zeiss Axio Imager, M2 microscope equipped with a Zeiss Axio-Cam MRC5 camera. Color images were acquired with Zeiss 33 software (v.4.8) using X100 is Cplan-NeoFLUAR 10X/0.3 and X400 is a Plan-APOCHROMAT 40X/0.95 objectives. Liver microscopy was performed using an Olympus BX51 microscope equipped with a Retiga 1300 camera (Q imaging). Color (12-bit) images were acquired with Bioquant 2012 software using UPlanApo objective (20×, numerical aperture.70). In lung and liver sections, neutrophil counts were performed based on the segmented morphology in high power fields (×40) in 30 measurements from 10 different randomly selected alveolar or portal areas, respectively.

### Plasma analysis

#### Measurements of endotoxin activity

Endotoxin activity in rat and human samples and in saline mixture of LPS with L-4F or Sc-4F was measured by limulus amoebocyte lysate (LAL) assay using kinetic-colormetric test (Endochrome-K, Charles River) in accordance with manufacturer protocol.

#### Measurement of plasma cytokines

The concentration of TNF-α and IL-6 in the human plasma/serum and the concentration of TNF-α, IL-6, and IL-10 in rat plasma was quantified by using commercially obtained ELISA kits (BD Biosciences) specific for humans and for rats, respectively.

#### Measurement of Myeloperoxidase

The Calbiochem® InnoZymeTM Myeloperoxidase Activity Kit (EMD Millipore) was used to quantify specific active human myeloperoxidase (MPO). The specific MPO Fluorometric Detection Kit (Enzo Life Science, Plymouth Meeting, PA. USA) was used to quantify total MPO in rat plasma. Measurements were performed in accordance with manufacturer's protocols.

#### HDL isolation

Human serum or plasma (50 µL) or rat plasma (50 µL) was combined with the magnesium/dextran sulfate reagent (5 µL, Pointe Scientific, Inc., MI, USA). The mixture was vortexed and incubated for 10 min at room temperature letting the reagent to precipitate the LDL and VLDL fractions, leaving the HDL fraction in solution. After centrifugation, HDL fraction was collected. In some human *in vitro* experiments, HDL fractions were collected by size exclusion chromatography using a Bio-Logic Fast Protein Liquid Chromatography system (Bio Rad) as previously described [31], [39].

#### Measurement of HDL-associated paraoxonase-1 activity

Paraoxonase-1 (PON1) activity was determined by adding HDL or HDL fractions separated by FPLC to the buffer containing paraoxon (Sigma-Aldrich), and measuring the rate of release of 4-nitrophenol at 405 nm as previously published [30], [31]. Briefly, 2-μL of serum or HDL fractions were added to 200 μL buffer (100 mmol/L Tris containing 2 mmol/L CaCl_2_, pH 8.0) containing paraoxon (1 mmol/L O, O-diethyl-O-p-nitrophenylphosphate), and the rate of release of 4-nitrophenol was determined spectrophotometrically. The assay was performed in a 96-well plate, and readings were taken every 2 minutes at 405 nm. The quantity of 4-nitrophenol formed was calculated from the molar extinction coefficient of 17 100 mol/L^−1^cm^−1^. One unit of PON activity was defined as 1 nmol of 4-nitrophenol formed per minute.

#### Measurement of serum and HDL cholesterol in humans

Total serum/plasma and HDL cholesterol was measured using commercial kit (Thermo Fisher Scientific Inc.).

#### Liver function test panel in rats

Plasma levels of alanine transaminase (ALT), aspartate transaminase (AST), alkaline phosphatase (ALP), total and direct bilirubin were measured by Animallab (Birmingham, AL) using ACE clinical chemistry system (Alfa Wassermann Inc.). Plasma triglycerides and total cholesterol were measured using corresponding commercial kits (Thermo Scientific).

#### Measurement of relative change of reactive oxygen species level in rat plasma

Relative change of reactive oxygen species level in plasma was measured using a 2′,7′-dichlorofluorescin (DCF, Sigma-Aldrich) assay. Rat plasma (5 µL) was diluted in 200 µL of PBS containing 50 µM DCFH and incubated in 96-well black plates for 1 h at 37°C in dark. Fluorescence was then measured with excitation and emission wavelengths of 485 nm and 530 nm, respectively, and then normalized by total plasma cholesterol.

#### Measurement of apoA-I level in human serum

Absolute values of apoA-I levels in patient's serum were measured using ELISA kit for human apoA-I (Mabthech USA) in accordance with manufacturer's protocol. These absolute apoA-I values were compared with relative apoA-I values measured based on immunobloting analysis similar to that described below, except the patient's serum and goat anti-human apoA-I antibody (Brookwood Biomedical, Birmingham, AL) were used. We found a very strong correlation (r = 0.95, P<0.0001, Pearson) between relative apoA-I levels measured based on WB data and absolute values measured by ELISA.

#### SDS Polyacrylamide Gel Electrophoreisis (SDS-PAGE); Immunobloting Analysis

Rat plasma aliquot (0.35 µl) was separated by 4–20% Tris-HEPES-SDS Polyacrylamide gel (Thermo) at 100 V and transferred to a nitrocellulose membrane (100 V, 2 h, 4°C). Following transfer, the membrane was blocked with TBS/3% gelatin for 1 hour and then washed three times (10 min each) with TBST (10 mM Tris pH 7.40, 150 mM NaCl, 0.1% Tween 20). The membrane was then probed with 0.1 µg/ml polyclonal HRP-labeled rabbit anti-mouse (cross reactive) apoA-I antibody (Brookwood Biomedical, Birmingham, AL) for 1 hour at room temperature, and then washed with TBST. The membrane was then incubated with 0.1 µg/ml strepavidin alkaline phosphatase (Bio Rad) with TBST/1%gelatin for 1h, followed by an additional wash with TBST first, and then with TBS. Color was developed with alkaline phosphatase kit (Bio Rad). Reaction was stopped by washing the membrane in water. After membrane was air dried, it was optically scanned and apoA-I bands were analyzed with Image-J Software (NIH, Bethesda, MD).

### Statistical Analysis

All results, unless otherwise specified, are reported as the mean ± SEM. Statistical analysis was performed using GraphPad Prism V.4.0.1 (GraphPad Software Inc.). Where appropriate, differences between groups were assessed by paired Wilcoxon signed rank test or unpaired Mann-Whitney test (for two experimental groups) or one-way ANOVA with post-hoc Bonferroni's Multiple Comparison test (for three or more groups). Pearson correlation was used for analysis of relationship between HDL-C, apoA-I, and PON1 activity in patient serum. Survival data was assessed by log-rank analysis (Kaplan-Meier survival method). Differences in survival at 24 h between groups in rat experiments were analyzed with Fisher's exact test. A value of P<0.05 was considered to be statistically significant.

## Results

### Effects of L-4F in endotoxemic rats

#### L-4F improves rat survival

**Figure 1:**
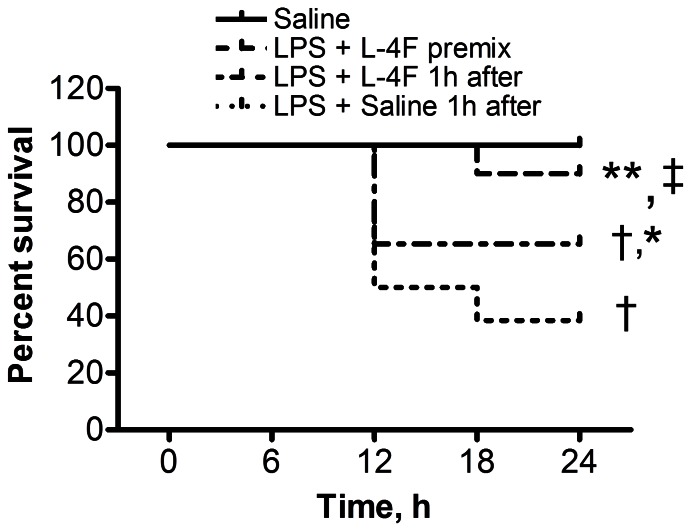
L-4F reduces mortality in endotoxemic rats. Rat survival at 24 h following LPS (30 mg/kg, i.p., n = 26) versus rats treated by L-4F (10 mg/kg) administered either simultaneously with LPS as a premix (i.p., n = 20) or one hour after LPS (L-4F given i.v., n = 26). Saline only (n = 10) did not get either LPS or L-4F. Log-rank analysis indicates a significant trend in rat survival changes among groups (*P*<0.005) *P<0.05 vs. LPS, †*P*<0.05 vs. Saline (sham), ‡*P*<0.05 vs. L-4F one hour post LPS.

Over a 24 h observation period, there was no mortality in the group of sham rats (Figure 1). In contrast, administration of 30 mg/kg LPS resulted in rat mortality, which occurred between the 12 and 24 h time points in all groups (Figure 1). Among endotoxemic rats, minimal survival rate at 24 h was observed in saline-treated rats (38%, *P*<0.001 vs. sham). L-4F, administered one hour post LPS injection, significantly improved rat survival to 65% (*P*<0.05 vs. LPS, *P*<0.05 vs. sham). When administered concurrently with LPS, L-4F virtually prevented rat mortality (90% survival rate, *P*<0.001 vs. LPS, NS vs. sham). There was a significant trend in rat survival changes among these groups (P<0.005, Figure 1).

#### L-4F inhibits ALI in endotoxemic rats

**Figure 2:**
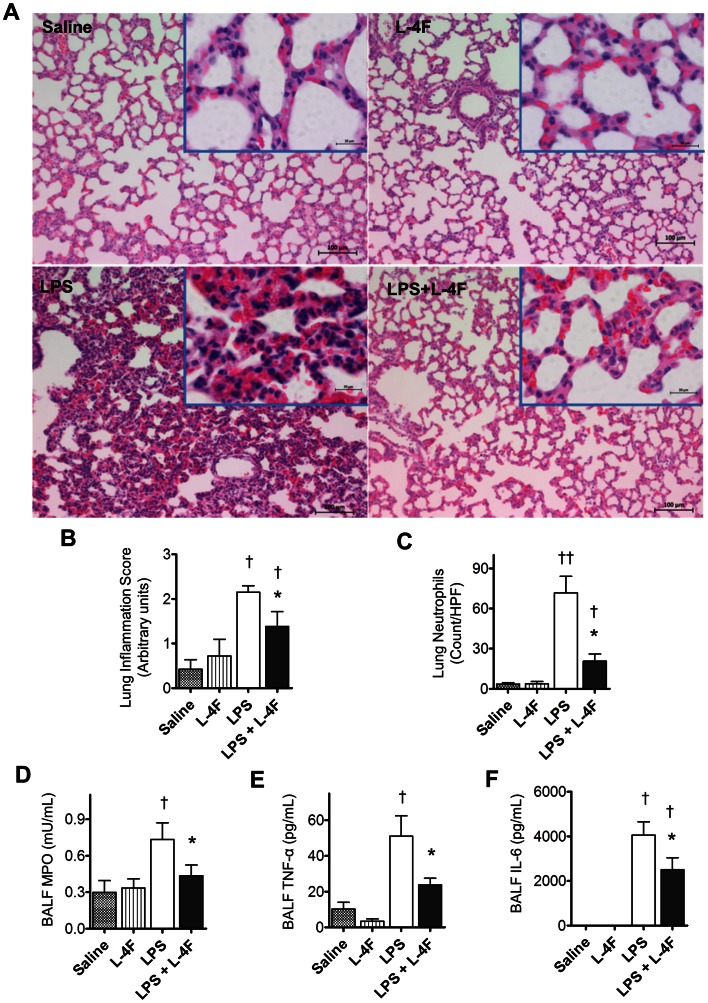
L-4F inhibits lung tissue inflammation in endotoxemic rats. **A:** Lung H&E stained sections (magnification ×100, the insert is a magnification ×630). In saline treated rats, the alveolar walls appear normal and do not contain extensive numbers of polymorphonuclear leukocytes (PMNs). In L-4F treated rats, lung tissue is similar to control, but some PMNs are identified in alveolar capillaries In LPS treated rats, the alveolar walls are thickened and contain congested capillaries with numerous PMNs. Some of these cells have infiltrated outside the capillaries. In rats treated with L-4F, the lung is less affected than the lungs of rats receiving LPS and is similar to the lungs which received L-4F alone. Scale bars, 100 µm (in inserts, 20 µm). **B:** Blinded analysis of lung inflammation score in 5–8 rat/group. **C:** Neutrophil counts in lung parenchyma. The vast majority of the neutrophils were found in the septae, and fewer neutrophils were in the alveolar spaces. Neutrophil counts were performed based on the segmented morphology in high power fields (×40) in 30 measurements from 10 different randomly selected portal areas (n = 5/group). **D, E, F:** BALF MPO, TNF-α, and IL-6 respectively (n = 4 for controls, n = 8 for LPS groups). *P<0.05 vs. LPS, †*P*<0.05 and ††*P*<0.01 vs. Saline and L-4F.

In order to assess pulmonary injury, L-4F was administered one hour post LPS injection in rats. After 6 h, rats were sacrificed, and lung tissues were stained with hematoxylin and eosin (H&E) and analyzed for inflammation (Figure 2A–C). Minimal inflammation was noted in lung tissues obtained from saline and L-4F-treated rats. In LPS-treated animals, the alveolar walls were thickened, and capillaries were congested with numerous polymorphonuclear leukocytes. Some of these cells infiltrated into the interstitial space. In endotoxemic rats treated with L-4F (1 h post LPS), the lungs were less affected and were similar to those of rats that received L-4F alone. Two experienced pathologists independently assessed inflammatory injury in lung tissue. L-4F decreased overall inflammation score (*P*<0.05 vs. LPS, Figure 2B, pathologist #1) and neutrophil counts in lung parenchyma (*P*<0.05 vs. LPS, Figure 2C, pathologist #2). Neutrophils were predominantly localized in the septae, and to a lesser extent in the alveolar spaces.

In BALF collected from LPS-treated rats, we also found increased levels of TNF-α, IL-6, and myeloperoxidase (MPO) (*P*<0.05 vs. controls, Figure 2D–F). L-4F inhibited pro-inflammatory cytokines and MPO elevation (*P*<0.05 vs. LPS).

#### L-4F inhibits liver inflammation and dysfunction in endotoxemic rats

**Figure 3:**
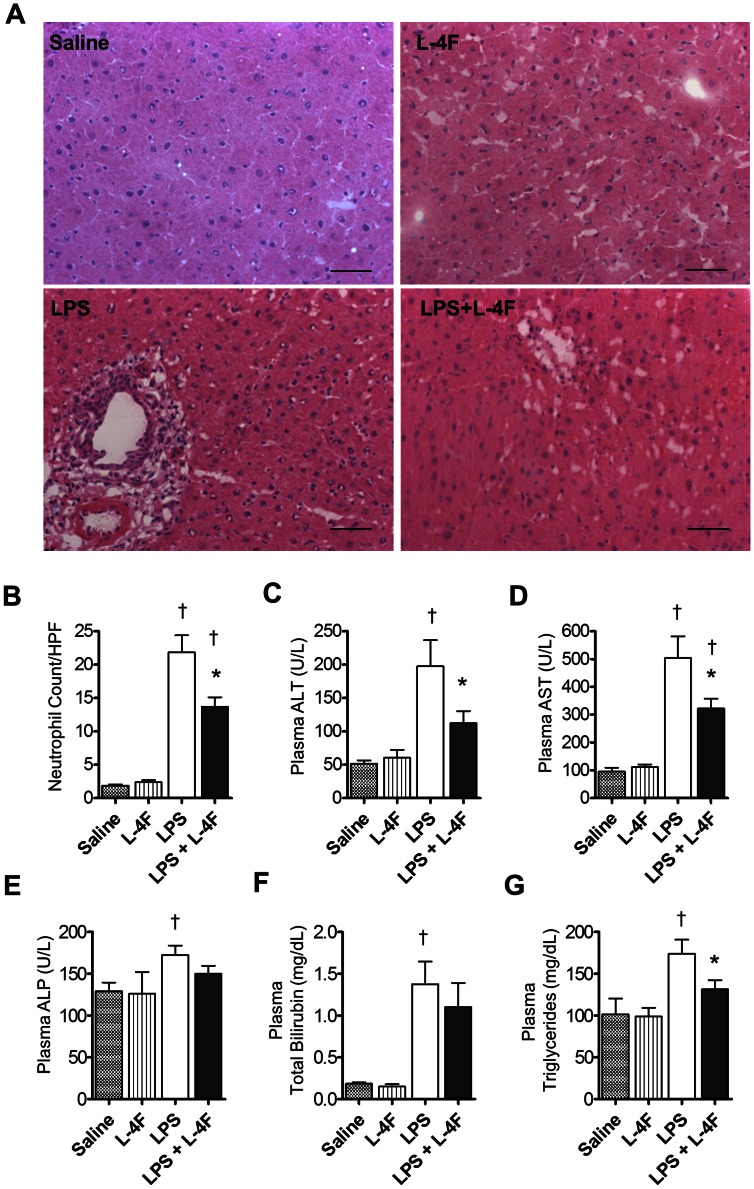
L-4F inhibits liver inflammation in endotoxemic rats. **A:** Liver sections (×20), stained with H&E. Liver sections from saline and L-4F-treated animals showed normal morphological and histological features without signs of inflammation or tissue damage. Liver sections from rats treated with LPS demonstrated significant infiltration of inflammatory cells, whereas liver sections from rats treated with LPS and L-4F had reduced infiltration of inflammatory cells. Scale bars, 50 µm. **B:** Blinded analysis of neutrophil infiltration in liver tissue. Neutrophil counts were performed based on the segmented morphology in high power fields (×40) in 30 measurements from 10 different randomly selected portal areas (n = 4/group). **C, D, E, F, G:** Plasma ALT, AST, ALP, Total Bilirubin, triglycerides, respectively (n = 8–12/group). *P<0.05 vs. LPS, †*P*<0.05 vs. Saline and L-4F.

Liver plays an important role in the pathogenesis of sepsis-induced ALI/ARDS [9]. We evaluated liver inflammation/injury at 6 h post LPS administration. H&E-stained liver sections from control groups displayed normal morphological and histological features (Figure 3A). Liver sections from rats treated with LPS alone showed tissue inflammation with a significant infiltration of inflammatory cells. In contrast, liver sections from endotoxemic rats treated with L-4F showed reduced inflammatory cell infiltration (*P*<0.001 vs. LPS, Figure 3B).

LPS-treated rats had increased plasma levels of alanine and aspartate transaminases, alkaline phosphatase, total bilirubin, and triglycerides (*P*<0.05 vs. saline, Figure 3C–G). L-4F decreased plasma transaminases and triglycerides levels in endotoxemic rats (*P*<0.05 vs. LPS).

#### L-4F inhibits systemic inflammation in endotoxemic rats

**Figure 4:**
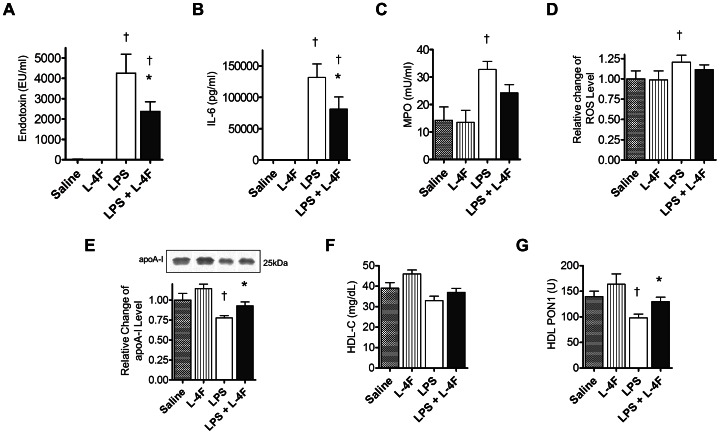
L-4F reduces systemic inflammation in endotoxemic rats. Plasma measurements taken at 6 h following LPS injection: **A:** Endotoxin levels; **B:** IL-6 concentration; **C:** MPO activity; **D:** Relative levels of reactive oxygen species as measured by DCF assay (normalized to plasma cholesterol); **E:** Western analysis of relative levels of apoA-I (based on plasma SDS-PAGE gel electrophoresis followed by immunobloting for apoA-I); **F:** HDL-cholesterol (HDL-C) levels; **G:** HDL-associated PON1 activity. *P<0.05 vs. LPS, †*P*<0.05 vs. Saline (n = 8 for controls, n = 12–14 for LPS groups).

Six hours post LPS administration, plasma endotoxin level was significantly elevated (*P*<0.05 vs. saline, Figure 4A). Similarly, plasma levels of IL-6, MPO and reactive oxygen species as measured by DCF assay was increased, whereas plasma levels of apoA-I decreased (*P*<0.05 vs. saline, Figure 4B–E). Although there was reduction in HDL cholesterol in LPS group, it was not statistically significant (Figure 4F). However, there was significant reduction in HDL-associated PON1 activity (*P*<0.05 vs. saline, Figure 4G). L-4F decreased plasma endotoxin level in LPS-treated rats and reversed the effect of LPS on IL-6, apoA-I, and PON1 activity (*P*<0.05 vs. LPS).

### Effects of L-4F *ex vivo* in the presence of LPS

#### L-4F inhibits LPS-mediated effects in human blood

**Figure 5:**
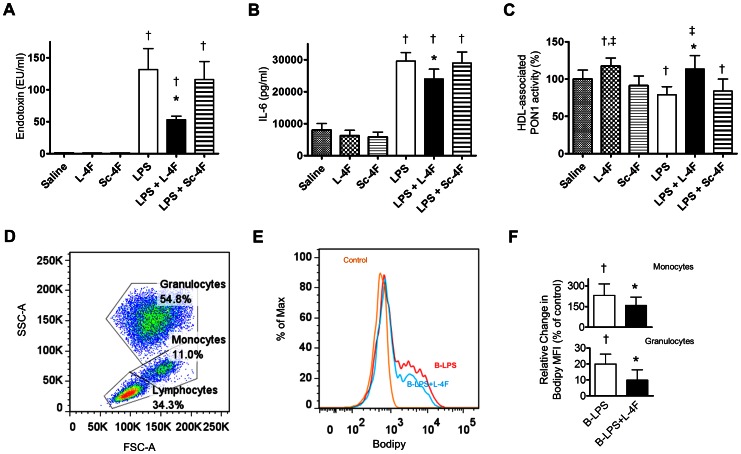
L-4F inhibits effects of LPS in human blood. **A, B, C:** L-4F (40 µg/ml), but not Sc-4F, decreases plasma endotoxin levels (**A**), plasma levels of IL-6 (**B**), and preserves HDL-associated PON1 activity after co-incubation with LPS (1 µg/ml) in the healthy whole human blood *ex vivo* (n = 6/group). **D, E, F:** L-4F inhibits LPS binding to leukocytes. Following incubation of Bodipy-LPS (B-LPS) for 30 min in human whole blood *ex vivo* (n = 5), fluorescence due to binding of B-LPS to leukocytes was measured using flow cytometry. **D:** A representative example of side scatter and forward scatter characteristics of leukocytes with major populations of lymphocytes, monocytes, and granulocytes isolated after incubation with either control (saline or L-4F), B-LPS (1 µg/ml), and B-LPS and L-4F (40 µg/ml). **E:** Cells selected as monocytes in (D) are plotted depending on intensity of Bodipy signal. **F:** Relative changes of Bodipy mean fluorescence intensity (MFI) compared to control MFI for monocytes and granulocytes are shown. *P<0.05 vs. LPS or B-LPS, †*P*<0.05 vs. Saline and L-4F alone.

We measured activity of L-4F against endotoxin-mediated inflammatory effects in whole human blood. LPS (1 µg/ml) was incubated in human blood for 12 h and was associated with high level of endotoxin activity in plasma (Figure 5A). LPS caused a strong inflammatory response, which was associated with high plasma IL-6 levels (Figure 5B) and decreased activity of HDL-associated PON1 (Figure 5C). L-4F, but not control peptide Sc-4F, significantly decreased endotoxin activity, inhibited plasma IL-6 elevation and preserved activity of HDL-associated PON1 (*P*<0.05 vs. LPS for all, Figure 5A–C).

#### L-4F inhibits LPS binding to leukocytes

Since LPS mediates many of its inflammatory effects *via* binding to leukocytes, we measured effects of L-4F on LPS binding/internalization by leukocytes in human blood using fluorescent Bodipy-LPS (1 µg/ml) and flow cytometry. Bodipy-LPS demonstrate biologic effects similar to LPS, which is inhibited by L-4F (data not shown). Leukocytes isolated from human blood were gated based on side scatter and forward scatter characteristics (Figure 5D). Monocytes, incubated with Bodipy-LPS, revealed a two-fold increase in Bodipy fluorescence compared to controls (*P*<0.05 vs. control cells, Figure 5E–F). A similar phenomenon was observed in granulocytes (Figure 5F). L-4F inhibited Bodipy-LPS-mediated fluorescence in each cell population (*P*<0.05 vs. Bodipy-LPS, Figure 5F).

#### L-4F binds LPS and decreases endotoxin activity

**Figure 6:**
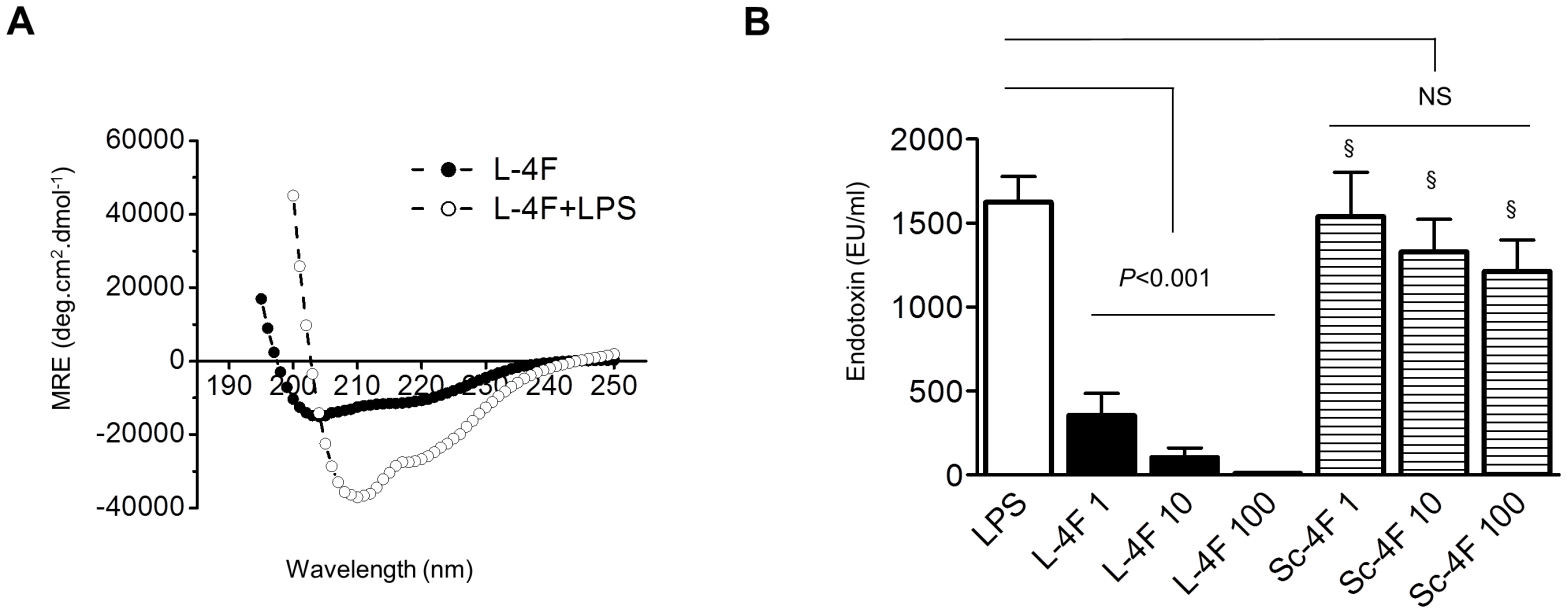
L-4F binds LPS and neutralizes endotoxin activity. **A:** Circular dichroism spectroscopy of L-4F 100 μg/ml alone (filled circles), and with LPS 1000 μg/ml (open circles) in PBS. An increase in the mean residue ellipticity (MRE) at 222 nm in the presence of LPS reflects the formation of L-4F-LPS complex. **B:** L-4F, but not Sc-4F, inhibits endotoxin activity of LPS in LAL assay. LPS (1 µg/ml) was mixed with L-4F or Sr-4F (1, 10, 100 µg/ml) and endotoxin activity was measured with kinetic colorimetric LAL assay (n = 3). §*P*<0.01 vs. L-4F.

A potential physical interaction between LPS and L-4F was suggested in our previous study as a mechanism to explain the peptide-mediated inhibition of LPS action [30]. To characterize the potential physical interaction purely between LPS and L-4F, we performed circular dichroism spectroscopy of L-4F, alone and in the presence of LPS, in PBS (Figure 6A). An increase in the mean residue ellipticity (MRE) at 222 nm in the presence of LPS (approximately by 150%) reflects higher helical content of L-4F due to the formation of LPS-L-4F complex.

To test whether LPS, bound to L-4F, decreases endotoxin activity, we incubated LPS (1 µg/ml) with L-4F (1, 10, and 100 µg/ml) in saline and assessed endotoxin activity using the limulus amebocyte lysate assay. L-4F reduced endotoxin activity in a dose-dependent manner (*P*<0.001 vs. LPS, Figure 6B). At the same concentrations, Sc-4F did not have significant effect on endotoxin activity.

### Effect of L-4F on inflammatory potential of ARDS patient serum *ex vivo*

#### ARDS patient physiological characteristics and survival

**Figure 7:**
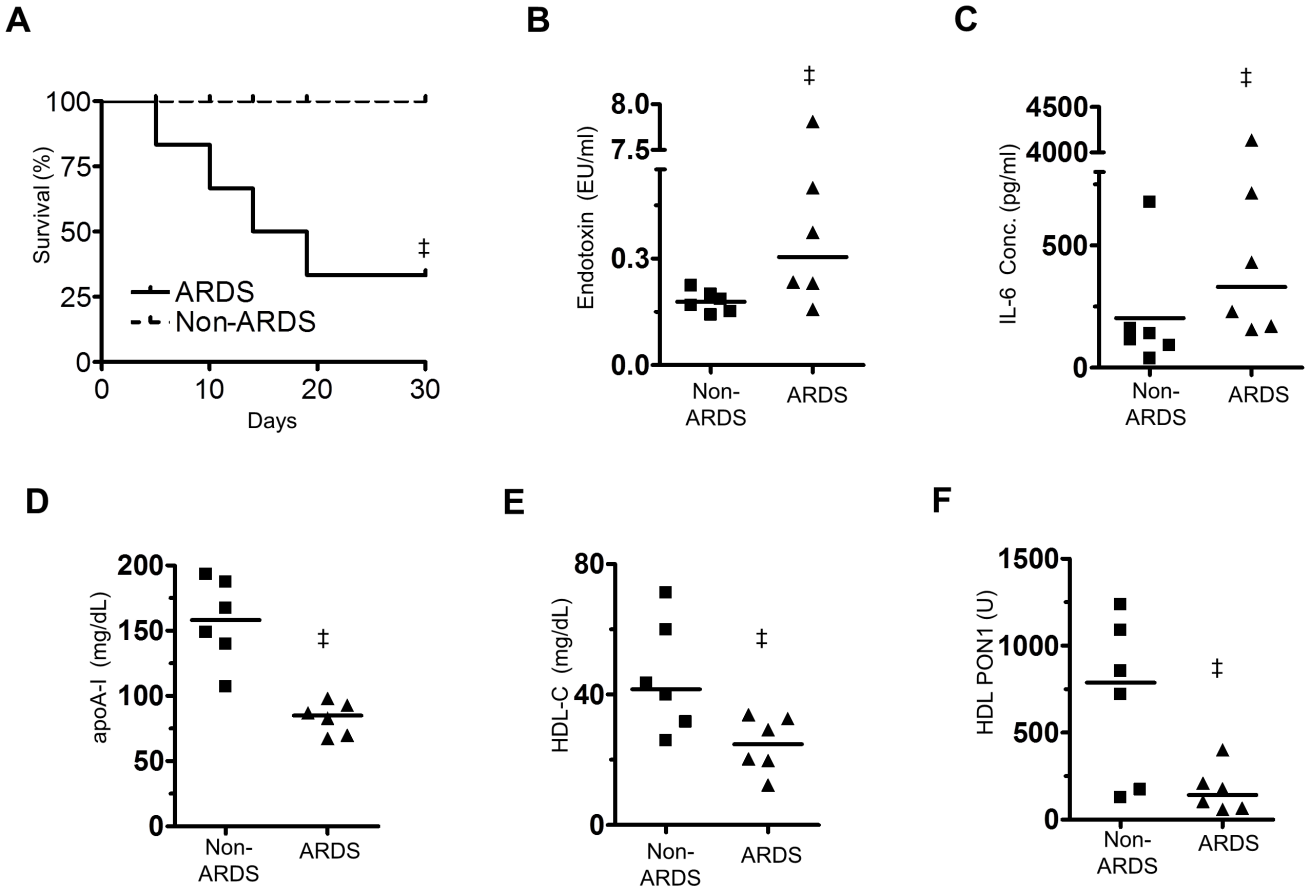
Survival rate and serum parameters of ARDS patients compared to non-lung disease (Non-ARDS) intubated control patients. **A:** Kaplan-Meier survival curve (n = 6/group). Four ARDS patients died within 30 days from admission to intensive care unit. **B, C, D, E, F:** Comparisons of serum endotoxin levels (**B**), levels of IL-6 (**C**), apoA-I (**D**), HDL-cholesterol (HDL-C) (**E**), and activity of HDL-associated PON1 (**F**) in ARDS and non-ARDS patients at admission to ICU. A horizontal line depicts the median. ‡*P*<0.05 vs. Non-ARDS.

Compared to non-lung disease intubated (non-ARDS) patients, ARDS patients had lower indices of respiratory function and higher acute physiology and chronic health evaluation scores (APACHE II) as shown in Table S1. Transaminase levels were higher in ARDS likely due to ongoing systemic inflammation related to sepsis (Table S1). Four ARDS patients (67%) died by 19 days after admission to ICU, whereas no non-ARDS patients died at least within 30 days (Figure 7A).

#### Septic inflammation markers in ARDS patient serum

On the day of intubation, ARDS patient serum had elevated levels of endotoxin activity and IL-6 compared to non-ARDS patients (1.551±1.253 EU/ml vs. 0.178±0.013 EU/ml, and 973.1±638.6 pg/ml vs. 203.1±96.17 pg/ml, respectively, *P*<0.05 for both, Figure 7B–C). ARDS patients also had lower levels of total serum cholesterol and PON1 activity than non-ARDS patients (81±7 mg/dL vs. 159±17 mg/dL, *P*<0.005, and 259±76 U vs. 1033±278 U, *P*<0.05, respectively). Serum apoA-I levels were lower in ARDS compared to non-ARDS patients (*P*<0.05, Figure 7D). HDL cholesterol (HDL-C) and HDL-associated PON1 activity (22±3 mg/dL vs. 41±6 mg/dL, *P*<0.05, Figure 7E, and 170±62 U vs. 700±183 U, *P*<0.05, Figure 7F, respectively) were also reduced.

A strong association between PON1 activity and levels of HDL-C and apoA-I has been shown in healthy humans [40]. In ARDS patients, we observed that PON1 activity (standardized to HDL-C level) was almost two-fold lower than in non-ARDS patients (7.9±2.3 vs. 14.5±3.6, NS). In non-ARDS patients, there was a strong correlation between levels of HDL-C, HDL-associated PON1 activity and apoA-I (r = 0.89, *P* = 0.016, and r = 0.88, *P* = 0.021, respectively, two tailed Pearson). No such relationship was found in ARDS. This suggests that ARDS is not only associated with reduced HDL, but also with pathologically remodeled HDL.

#### L-4F inhibits neutrophil activation induced by serum from ARDS patients

**Figure 8:**
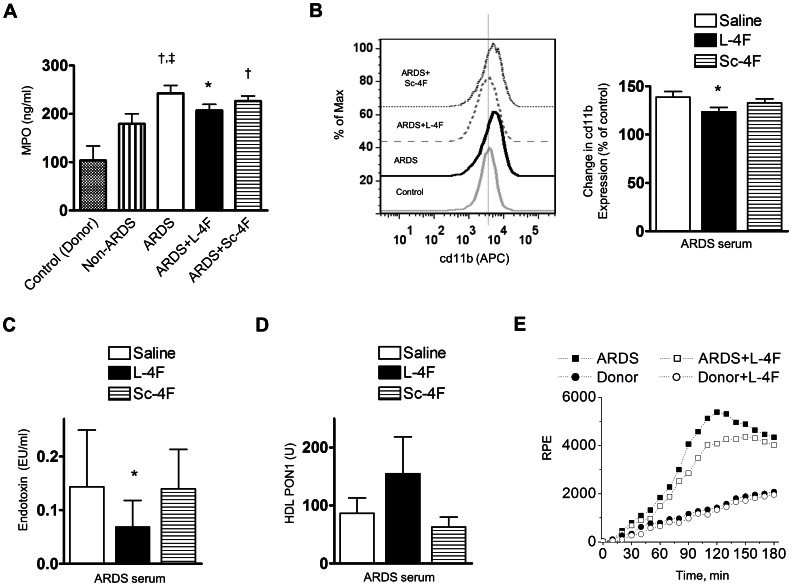
L-4F inhibits inflammatory potential of ARDS serum on human cells. In **A, B, C, D:** human neutrophils isolated from healthy individuals (Control) were incubated in ARDS or non ARDS patient serum for 1h in the presence of 40 µg/ml L-4F or Sc-4F (n = 5). **A:** Change in MPO levels in cell media (ARDS and non-ARDS serum). **B:** Measurements of cd11b expression in neutrophils stained with anti-human cd11b (activation epitope) allophycocyanin (APC). A representative histogram of surface expression of cd11b for different treatments (left); statistical data presented as percent changes in cd11b expression (based on mean fluorescent intensity) relative to the saline control (right). Vertical dashed line represents median cd11b activation in control. **C:** Change in endotoxin levels in ARDS serum. **D:** Change in HDL-associated PON1 activity in ARDS serum. **E:** L-4F (40 μg/ml) inhibits superoxide formation by leukocytes mediated by ARDS serum. Superoxide formation was measured with lucigenin-amplified chemiluminescence in freshly isolated human leukocytes at room temperature. A representative example of six consistent experiments is shown. RPE  =  Relative Photon Emission. *P<0.05 vs. saline-treated LPS or ARDS, †*P*<0.05 vs. control groups, ‡*P*<0.05 vs. non-ARDS.

Neutrophils are major mediators of ARDS/ALI. Freshly isolated human neutrophils were incubated with non-ARDS and with ARDS patient serum, and the efficacy of L-4F (40 µg/ml) in reducing the inflammatory potential of ARDS serum was tested. Neutrophil activity was assessed by measuring the MPO level in cell media. Only ARDS serum induced a significant increase in MPO levels (*P*<0.05 vs. control and non-ARDS), which was inhibited by L-4F but not by Sc-4F (Figure 8A). We also measured changes in the expression of cd11b on neutrophil membranes, a marker of neutrophil activation using flow cytometry (Figure 8B). ARDS serum increased cd11b expression by 39% compared to non-ARDS controls (Figure 8B). L-4F added to ARDS serum reduced cd11b expression by approximately 40% (*P*<0.05, vs. ARDS), whereas Sc-4F had minimal effect. These effects of L-4F were accompanied by a reduction in endotoxin activity in ARDS serum (*P*<0.05 vs. saline-treated ARDS, Figure 8C).

#### L-4F inhibits superoxide formation by leukocytes induced by ARDS patient serum

Oxidative stress is important in the pathogenesis of ARDS/ALI. Freshly isolated human leukocytes were incubated in ARDS patient serum, and superoxide formation as a marker of oxidative stress was measured using lucigenin-amplified chemiluminescence. Leukocytes incubated in ARDS serum caused stronger chemiluminescence than leukocytes incubated in healthy serum (Figure 8E). L-4F in ARDS samples inhibited the rate as well as peak chemiluminescence emission. L-4F produced similar effects in all ARDS samples resulting in decreased superoxide formation (*P*<0.05 vs. ARDS, n = 6).

## Discussion

Treatment options for ARDS patients are limited and generally rely on supportive therapies to maintain adequate oxygenation and hemodynamic status. Despite the recognition that HDL and apoA-I modulate immune cell function, their role in reducing complications associated with ARDS is not clear. Herein, we demonstrate that the 18 amino acid residue apoA-I mimetic peptide L-4F, which bears a similar class A amphipathic structure as that found in helical repeats of apoA-I, interacts with LPS and prevents activation of leukocytes or neutrophils by LPS and septic ARDS serum. In a clinically relevant model, L-4F inhibits lung and liver injury and improves mortality in rodents by inhibiting endotoxin activity and by improving the functional properties of HDL. Our study therefore provides the rationale for the clinical testing of the L-4F peptide in sepsis-associated ARDS.

Several mechanisms are involved in beneficial effects of L-4F in our study. First, L-4F directly binds to and neutralizes LPS. Formation of L-4F-LPS complexes has been suggested by us previously [30]. Here we demonstrate that L-4F can directly interact with LPS. We also demonstrate that L-4F directly decreases the endotoxin activity of LPS. LPS mediates many of its inflammatory effects *via* binding to leukocytes and activating Toll-like receptors [2], [3]. Further, we demonstrate that L-4F decreases LPS binding to leukocytes. This effect is likely mediated by high affinity of L-4F to LPS. L-4F competes with lipopolysaccharide-binding protein for LPS binding as shown by us previously [30].

Another mechanism of L-4F is via its effects on HDL. In serum of sepsis-mediated ARDS patients, we found high levels of endotoxin and low levels of apoA-I and HDL. Increasing plasma apoA-I or HDL has been shown to reduce endotoxemia-associated complications [24], [26], and so did L-4F in our present study and in previous experiments [31]. HDL neutralizes LPS by binding to LPS and mobilizing LPS to the liver for its degradation and excretion [14], [23], [24]. It has been suggested that L-4F facilitates LPS binding to HDL in endotoxemic rats [31]. In addition, HDL via apoA-I down-regulates CD14 and CD11b expression in monocytes and neutrophils [23], [41]. L-4F has been recently shown to produce similar effects in human monocyte-derived macrophages [42] and down-regulates cell-surface TLRs in these cells [43].

We also found that the structural and functional integrity of HDL in ARDS was compromised. In our rodent model of ARDS, plasma apoA-I was severely reduced. LPS induces acute inflammation/injury in rat liver which is the major site for apoA-I biosynthesis [44]. Reduction of apoA-I synthesis in the liver likely occurs through LPS-mediated NF-κB activation [45]. L-4F treatment significantly reduces hepatic inflammation. Further, apoA-I is a selective target for MPO-catalyzed oxidation, which leads to pro-inflammatory HDL [46]. LPS causes an increase in MPO activity which is prevented by L-4F. L-4F and apoA-I share structural and functional properties, it has been recently shown that 4F can serve as a reactive substrate for MPO-derived hypochlorous acid [47].

ARDS is associated with increased oxidant stress. HDL is a major antioxidant due to presence of apoA-I and PON1 as its constituents [20], [48]. PON1 protects lipoproteins and plasma membranes against oxidation by hydrolyzing lipid peroxides 49,50, and reduces oxidative cytotoxicity [20]. PON1 levels and activity is reduced in acute inflammatory state, which decreases overall HDL antioxidant activity [20] and leads to inability of acute-phase HDL to protect against lipid oxidation [18], [20]. Our results indicate that L-4F preserves HDL-associated PON-1 activity and therefore facilitates PON1-mediated reduction in lipid hydroperoxides. Other investigators including a murine model of asthma have previously demonstrated the ability of L-4F to promote anti-inflammatory effects of HDL, including increases in PON1 [51]–[53].

In the current study, we found significant mortality reduction in L-4F-treated rats even when it is administered 1 hour post LPS. This is associated with significant reduction in hepatic and pulmonary inflammation. Similar to our results, another recent study also found improvement in mortality and ALI after L-4F treatment in endotoxemic rats [54]. This was associated with stimulation of pulmonary sphingosine-1-phosphate receptor 1, activation of Akt and down regulation of NF-κB pathway [54]. Our data though suggest that major protective mechanisms of L-4F in LPS-mediated inflammation and ALI in this [54] and our models include inhibition of endotoxin activity by direct interaction with the bacterial lipopolysaccharide along with a preservation of HDL function.

Our study has a number of limitations. The rodent model of LPS-mediated endotoxemia reproduces only partially the pathophysiological mechanisms of septic inflammation and organ injury (including ALI/ARDS) induced by gram-negative bacteria in humans [55]. Nevertheless, LPS-related model is extensively used and could be considered as fast and reliable approach for the “proof-of-concept” or new drug screening. Another limitation is related to short period of follow-up in mortality study. The death of L-4F-treated rats could hypothetically be delayed. In our preliminary experiment (n = 6/group) with 10-days follow-up, no rat died after 24 h post LPS in saline-treated and L-4F-treated groups. This is consistent with the results of a recent 72-hours follow-up study performed in relatively similar conditions [54]. In the present study, we used only single injection of L-4F. Since L-4F is non-toxic and rapidly metabolized in the organism, repeated L-4F administrations in septic animals would potentially produce even better anti-inflammatory effects and this option could be tested in future research. Another study limitation is related to lack of Sc-4F treatment group in our *in vivo* experiments. However, a number of laboratories reported the failure of scrambled form of 4F to inhibit effects of LPS in *in vivo* experiment [31] as well as to reproduce anti-inflammatory and anti-oxidant effects of 4F in apoE null mice [56] and in murine model of asthma [53].

Despite an improved understanding of ARDS over the past 30 years, the impact on modulating disease-related outcomes has been relatively modest [57]. To date, the only therapy which has shown impact on ARDS-related mortality has been low-tidal volume ventilation [58]. Current 60-day mortality rates for ARDS patients are approximately 35%, although those individuals with sepsis as an underlying cause demonstrate increased mortality rates (45–60%) [59]. Similarly, our cohort of sepsis-related ARDS patients demonstrated increased mortality despite an appropriate ventilator strategy. For these individuals, the need for a specific disease-modifying therapeutic compound is critical, and L-4F peptide may be one such potential agent. Acceptable safety profile for paranteral and oral forms of 4F has been recently shown in selected patient groups with high-risk cardiovascular disease [60], [61]. Using L-4F in ARDS would likely provide beneficial effects on both inflammatory and oxidative pathogenic pathways of ARDS. Our data showing improvement in ALI indices and survival in endotoxemic rats along with the successful *ex vivo* LPS neutralization in ARDS patient serum by L-4F peptide strongly suggest the therapeutic potential of this peptide in early sepsis-related ARDS.

## Supporting Information

Table S1 **Patients characteristics on admission to ICU.**
(TIF)
