# Supplementary figures and images for: Anti-Inflammatory Mechanisms of Apolipoprotein A-I Mimetic Peptide in Acute Respiratory Distress Syndrome Secondary to Sepsis
Source: PLoS One. 2013 May 14;8(5):e64486. doi: 10.1371/journal.pone.0064486 (PMC3653907; doi:10.1371/journal.pone.0064486)

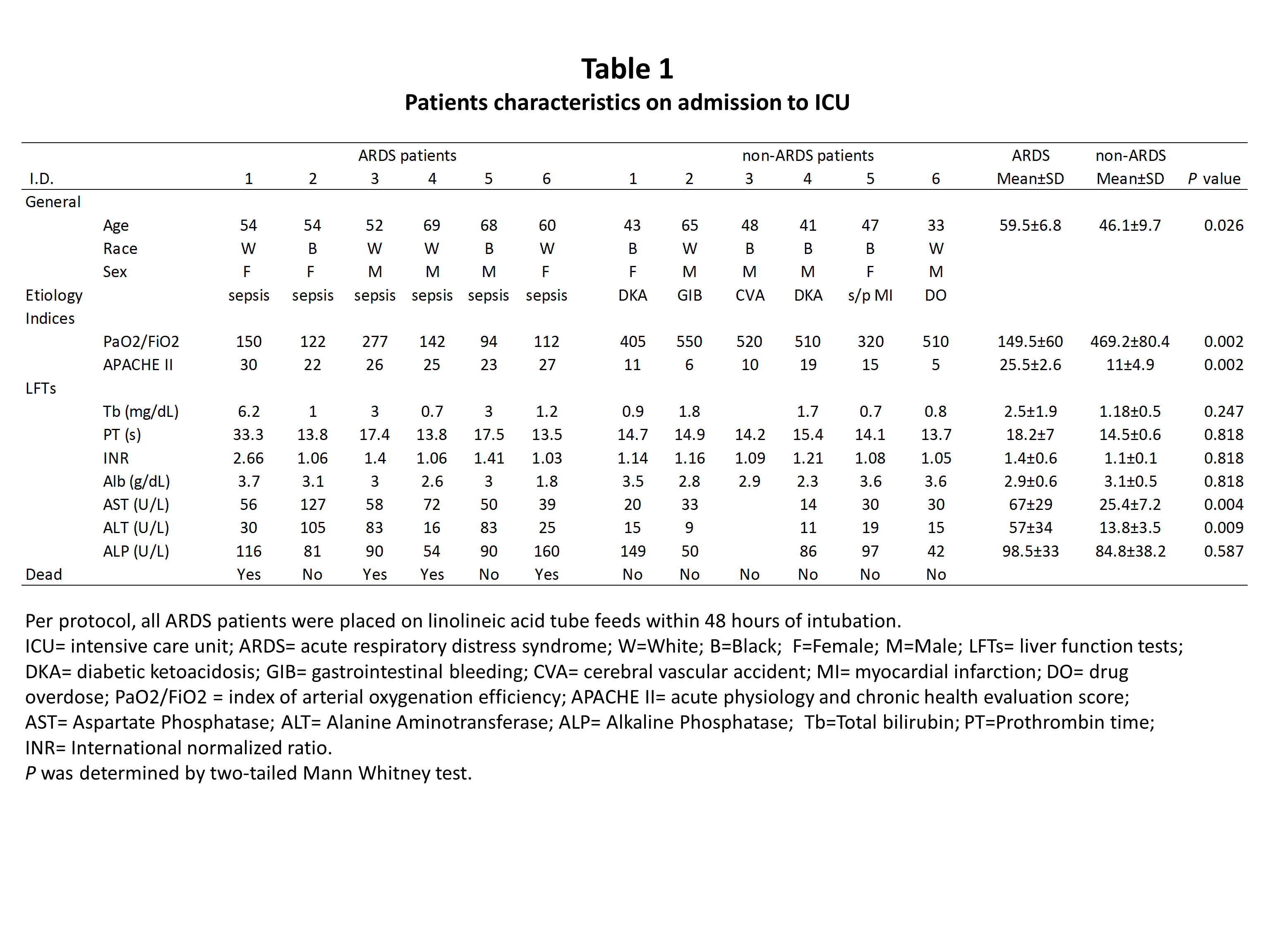

Supplement: Table S1 — Patients characteristics on admission to ICU. (TIF) [file pone.0064486.s001.tif]
